# Building National Capacity for Dementia Caregiving Research: The NIA Edward R. Roybal Centers

**DOI:** 10.1093/geroni/igaa057.1887

**Published:** 2020-12-16

**Authors:** Karina Davidson, Lisa Onken

## Abstract

More than 5.8 million Americans are currently living with Alzheimer’s disease and related dementias, and they are cared for by over 16 million people at an estimated annual cost of $290 billion. The need for innovative, evidence-based interventions to support these patients and caregivers is critical to addressing this problem. The goal of the NIA Edward R. Roybal Centers for Translational Research in the Behavioral and Social Sciences of Aging is to translate and integrate basic behavioral and social research findings into interventions aimed at innovatively improving both the lives of older people and the capacity of institutions to adapt to societal aging. The Roybal Centers will develop research within the conceptual framework of the multidirectional and translational NIH Stage Model to produce these implementable, principle-driven behavioral interventions. The program has now funded new Centers, and each has a unique focus. Areas of concentration include promoting caregiving mastery (Emory University), integrating the use of technology in care support to improve assessments and interventions in care provision (Oregon Health & Science University), developing behavioral interventions to reduce isolation and promote social connectedness in caregivers (University of Rochester), promoting health in racial/ethnic minorities (University of Illinois at Chicago), and using insights from data science and behavioral economics to improve palliative care delivery and long-term support facilities for persons with dementia and their caregivers (University of Pennsylvania). Center leaders will present an overview of their cutting-edge, early-stage research projects and discuss implications for improving care of caregivers and patients.

